# Comparing Compensation of U.S. Military Physicians and Civilian Physicians in Residency Training and Beyond

**DOI:** 10.7759/cureus.12931

**Published:** 2021-01-27

**Authors:** Sharon K Stortz, Lisa M Foglia, Andrew S Thagard, Barton Staat, Monica A Lutgendorf

**Affiliations:** 1 Obstetrics and Gynecology, U.S. Naval Hospital (USNH) Okinawa, Okinawa, USA; 2 Obstetrics and Gynecology, Maternal Fetal Medicine, Womack Army Medical Center, Fort Bragg, USA; 3 Obstetrics and Gynecology, Maternal Fetal Medicine, Naval Medical Center Portsmouth, Portsmouth, USA; 4 Obstetrics and Gynecology, Maternal Fetal Medicine, Uniformed Services University, Walter Reed National Military Medical Center, Bethesda, USA; 5 Obstetrics and Gynecology, Maternal Fetal Medicine, Naval Medical Center San Diego, San Diego, USA

**Keywords:** military physician pay, military tax advantage, graduate medical education, military physician

## Abstract

Introduction

Resident physicians have a professional degree but are compensated less than other recently graduated professionals such as lawyers or nurse practitioners. The U.S. Military Healthcare System differs from the civilian setting in that physicians' salaries are based primarily on military rank. We compared military and civilian physician salaries across various specialties to determine if the increased military pay during residency compensates for military attending physicians' lower income as compared to their civilian counterparts.

Methods

This cross-sectional study compares military and civilian pay for resident and attending physicians in the fields of Obstetrics & Gynecology (OB/GYN), Family Medicine, and General Surgery. Military pay was obtained from 2018 Defense Finance and Accounting Service (DFAS) data. Civilian salaries were obtained from the Medscape 2018 Residents Salary & Debt Report, Medical Group Management Association (MGMA) 2018 Provider Compensation Report, and 2017-2018 Association of American Medical Colleges (AAMC) Faculty Salary Report.

Results

Military resident physicians earned 53% more than civilian residents while military attending physicians earned 32%-58% less (after taxes) than their civilian counterparts, varying by specialty. Military attending physicians' negative pay differential occurred in both academic and non-academic practice environments through MGMA data.

Discussion

The positive pay differential in military residency does not compensate for the negative pay differential of military attending physicians face as compared to their civilian counterparts. This negative pay differential persisted when comparing post-tax pay. Some military service benefits, such as decreased educational debt, are challenging to quantify and vary considerably between individuals. As the military seeks to reshape its healthcare force, military and civilian compensation differences should be considered.

## Introduction

Adequate healthcare access is an important national priority, with the U.S. Government supporting Graduate Medical Education (GME) at over 16 billion dollars in 2015 [1]. Though there is some variation in physician post-graduate salaries based on specialty and year in training, residency training generally involves long work hours and low salaries. Resident physicians earn significantly less than other post-graduate professionals, with an average resident salary of $59,300 annually in 2018 [2] as compared to other professions such as lawyers; in 2017, the reported median base salary for newly graduated lawyers was $115,000 annually, or $2,211 weekly [3], for firms with 51-100 lawyers. In 2002, a group of physicians filed an anti-trust lawsuit against the Association of American Medical Colleges (AAMC), alleging a collusive agreement and unfair practices [4]. Though ultimately dismissed [5], the lawsuit brought up many concerns of resident physicians, including the long work hours, low wages, and inability to negotiate for better working conditions or training opportunities.

GME in the U.S. military is unique because resident physicians earn salaries commensurate with their military rank, exceeding the resident salary in the civilian setting. Additionally, military resident physicians receive the same benefits as other military personnel such as housing and sustenance allowances. Differences in military residency pay could influence resident decisions on entering GME training in the military setting and could impact retention and recruiting. Although military attending physicians earn salaries and allowances based on their military rank, specialty bonus, and, in some instances, retention bonuses, they tend to earn less money than their civilian peers in the same specialties [6]. We sought to compare salaries between military and civilian physicians, at the trainee and attendee levels, to determine if the positive pay differential for military residents compensates for the negative pay differential of military attending physicians as compared to their civilian counterparts. Amid recent initiatives to reshape military medicine, we believe this question is important in discussing the costs, and retention, of military physicians.

## Materials and methods

This study is a cross-sectional study comparing aggregate resident and attending physician salary data for Obstetrics & Gynecology (OB/GYN), Family Medicine, and General Surgery, selected to represent a broad range of specialty information. Military pay was derived from the 2018 Defense Finance and Accounting Services (DFAS) tables. Values collected included base pay, housing/subsistence allowances, incentive, and board certification pay. The median housing allowance from all military training sites was calculated, and the housing allowance was assumed to be 'with dependents.' Compensation for military residents was the rank of O-3 with over two years of service, which corresponds to a third-year resident (post-graduate year three, or PGY-3). Compensation for civilian residents was obtained from the 2018 Medscape Resident Salary and Debt Report national average salary for PGY-3 residents [2].

Attending salaries were calculated as 'junior attending' and 'senior attending.' 'Junior attending' corresponds to a military rank of O-4 with over six years of service to approximate the period of the first three years of post-residency training. 'Senior attending' corresponds to a military rank of O-5 with over 10 years of service, reflecting the period four to seven years after graduation from residency. Housing allowance calculations were based on the median allowance from all military training locations per rank category. Specialty pay for attending physicians was obtained from DFAS 2018 data [7] and varied by specialty; bonus payments were $8,000 annually for residents and an additional $6,000 annually for attending physicians receiving board certification pay. Since many military physicians enter service through the Uniformed Services University of the Health Sciences (USUHS), DFAS pay scales for the rank of O-1 were used to calculate the pay medical students at USUHS earn during medical school.

Attending salaries were obtained from the Medical Group Management Association (MGMA) 2018 Provider Compensation Report [6] (reports all practice settings, used with permission). This data includes total Medicare wages, bonuses or incentive payments, research stipends, honoraria, 401K, life insurance, and any other pre-tax deductions. MGMA compensation salaries do not reflect expense reimbursements, fringe benefits, flex spending accounts, health insurance, or employer contributions. Data were derived from the 2018 Association of American Medical Colleges (AAMC) Faculty Salary Report [8] for academic attending physicians. Academic salary numbers originated from summary statistics for all public and private institutions schools for the Associate Professor rank. Descriptive data are reported for salaries in all three specialties. The pay gap was calculated using non-subspecialty salaries for OB/GYN, General Surgery, and Family Medicine with Obstetrics. Marginal tax rates published by the U.S. Internal Revenue Service (IRS) for the year 2020, instead of 2018, were used to calculate federal taxes [9] due to tax code changes in 2018 that changed the standard deduction amount, making 2018 tax less applicable to current incomes. The state of Virginia was selected to be a representative state for calculating state taxes [10] due to the large population of military physicians stationed there. For tax calculations, we assumed the physician was married with a family of four, filing jointly, and taking the standard deduction of $24,800.

## Results

Military resident pay was $88,694, calculated as the rank equivalent of PGY-3, with a salary of $56,364 annually and an additional $8,000 for annual incentive pay, housing allowance ($21,276 yearly), and subsistence allowance ($3,054 yearly). Civilian PGY-3 residents earned an average salary of $57,000, giving military residents a positive 35% differential. Military attending physicians receive a specialty-specific bonus ranging between $43,000 and $54,000 annually. Using the median housing allowance of all training sites, the senior military attending physician's total compensation was $177,204, $175,204, and $166,204, respectively, for OB/GYN, General Surgery, and Family Medicine (Table 1).

**Table 1:** Military attending physician pre-tax pay and allowances, in U.S. Dollars BAS = basic allowance for sustenance; BAH = basic allowance for housing (median allowance for all training sites); IP = incentive pay. BC = board certified pay

Pay and allowances	OB/GYN	General Surgery	Family Medicine
Junior staff base pay	$74,862	$74,862	$74,862
Senior staff base pay	$89,208	$89,208	$89,208
BAS (yearly total)	$3,048	$3,048	$3,048
Junior staff BAH (yearly total)	$23,346	$23,346	$23,346
Senior staff BAH (yearly total)	$24,948	$24,948	$24,948
IP (1-year rate)	$54,000	$52,000	$43,000
BC pay	$6,000	$6,000	$6,000
Total compensation			
Junior staff	$161,256	$159,256	$150,256
Senior staff	$177,204	$175,204	$166,204

OB/GYN senior military attending physicians experienced a negative pay differential of 45% (-$145,696) compared to median attending civilian salaries of $322,900 as reported by MGMA. Compared to civilian academic physicians, military OB/GYN attending physicians had negative differentials of 38% and 40% for junior and senior staff, compared to Assistant Professor and Associate Professor, respectively, as reported by the AAMC. Military attending physicians in General Surgery and Family Medicine also had negative pay differentials, with 58% (-$239,942) and 32% (-$76,728), respectively, as compared to MGMA data. As with OB/GYN salaries, the negative pay differential for general surgeons and Family Medicine physicians persisted compared to academic physician salaries. Junior and senior military attending general surgeons had negative differentials of 52% and 57%, respectively ($171,744 and $228,796) than Assistant Professors and Associate Professors as reported by the AAMC. For Family Medicine physicians, this same comparison of military and academic attending physicians yielded negative differentials of 25% (-$50,744) and 22% (-$47,796), respectively (Table 2).

**Table 2:** Comparison of military and civilian pre-tax compensation in U.S. dollars

Pay (source of data for civilian pay)	Military	Civilian	Difference	% difference
OB/GYN				
Junior Staff/Assistant Prof (AAMC)	$161,256	$258,000	-$96,744	37.5%
Senior Staff/Associate Prof (AAMC)	$177,204	$295,000	-$117,796	39.9%
All Practices (Senior mil) (MGMA)	$177,204	$322,900	-$145,696	45.1%
General Surgery				
Junior Staff/Assistant Prof (AAMC)	$159,256	$331,000	-$171,744	51.9%
Senior Staff/Associate Prof (AAMC)	$175,204	$404,000	-$228,796	56.6%
All Practices (Senior mil) (MGMA)	$175,204	$415,146	-$239,942	57.8%
Family Medicine				
Junior Staff/Assistant Prof (AAMC)	$150,256	$201,000	-$50,744	25.2%
Senior Staff/Associate Prof (AAMC)	$166,204	$214,000	-$47,796	22.3%
All Practices (Senior mil) (MGMA)	$166,204	$242,932	-$76,728	31.6%

**Figure 1:**
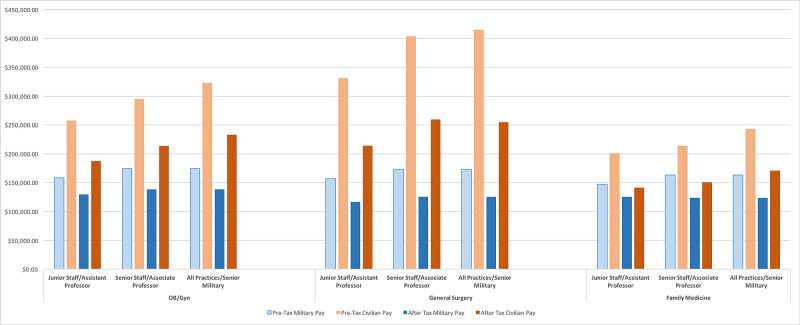
Military and civilian attending physicians compensation, in U.S. Dollars

Housing and sustenance allowances for military members are non-taxable, providing a tax advantage compared to civilian counterparts. Because of this tax advantage, post-tax salaries for civilian and military staff were calculated and compared to evaluate for changes to pay differentials (Figure 1). When federal and state taxes (using the federal marginal tax rate and Virginia state income tax rates) were calculated, the negative pay differential for military staff physicians persisted. For military junior attending general surgeons, the pre-tax pay differential was negative 52% while the post-tax differential was negative 46%. The pre-and -post-tax differentials for OB/GYN junior military attending physicians were negative 38% and negative 31%, respectively. For family practice junior military attending physicians, the differential was negative 26% and negative 21% for pre-and -post-tax compensation. The negative post-tax differentials persisted for senior military physicians when comparing their salaries to senior academic physicians and physicians in all practice environments using MGMA data (Figure 1). 

## Discussion

Civilian resident physicians earn comparatively lower salaries than other professionals upon graduation. Military resident physicians earn higher salaries than their civilian counterparts by 53% (post-tax pay), which is a substantial difference in annual salary. Our analysis demonstrates that a military resident completing a four-year residency would earn $119,244 more throughout their training than a comparable civilian; however, the civilian OB/GYN attending physician surpasses this differential in the first two years of practice. While the tax advantage of non-taxable military allowances is often cited as compensating for lesser overall pay than civilian physicians, our calculations show the negative pay differential for military physicians persists, likely due to the relatively small percentage of a military physician's salary that is non-taxable (housing and subsistence allowances are approximately $28,000 annually). Our calculations question the often-held belief that military pay's tax advantages help close the pay gap between military and civilian physicians.

However, a negative pay differential is not the only factor to consider when evaluating attending physician pay disparities. Medical school debt is a significant financial concern, with 67% of civilian physicians reporting $50,000-100,000 of debt in the first two years post-medical school [2]. Though we cannot measure medical school debt for military physicians, many military physicians were recipients of the Health Professions Scholarship Program (HPSP) or graduated from the Uniformed Services University of the Health Sciences (USUHS), both of which cover medical school tuition. USUHS also provides a salary while in medical school, so military physicians likely have minimal medical school debt than civilian physicians. However, both HPSP and USUHS carry a service obligation, ranging from two to four years for HPSP and seven years for USUHS graduates. For a USUHS graduate with this obligation, seven years of post-tax military pay (calculated as six years at military junior staff and one year at senior staff) will be $918,785 as compared to $1,357,777 earned in the civilian setting (post-tax pay, six years at civilian assistant professor salary, and one year at all practices salary), for a deficit of $438,992. This total does not include the salary medical students receive at USUHS (the rank equivalent of O1 with a base pay of $3,107 and housing allowance of $2,205). Including post-tax salary and benefits, approximately $261,175 is earned during medical school. When added to the calculated residency and seven-year military service, the total earned as a military physician who attended USUHS is $1,402,770 as compared to $1,461,345 a civilian physician earned during residency and seven years post-residency (a deficit of $58,575). This calculation does not take into account medical school debt for civilian physicians. In 2018, almost one-quarter of residents reported no medical school debt, one quarter reported $200,001-$300,000 of medical school debt, and almost a quarter reported over $300,000 of medical school debt [2]. A USUHS graduate would likely break even with about 50% of civilian graduates due to the burden of medical school debt.

Many military compensations and retention factors are difficult to account for such as free universal health insurance and no malpractice insurance fees. It is even more challenging to account for the possibility of retirement savings and investment opportunities that may exist for military physicians who do not have medical school debt. We did not include military Multiyear Specialty Pay into our calculations, as physicians are only eligible for this pay after their initial service obligation is complete, and accepting the pay incurs additional military obligation. While prior studies have demonstrated that debt avoidance results in higher net present value (NPV) in primary care and other relatively low paying specialties, higher-paying specialties, such as orthopedic surgery, can more quickly close the salary gap between public and private salaries [11]. Although future earning potential can offset medical education's high costs, primary care specialties earning lower annual salaries in the civilian setting never achieve the same NPV as colleagues that receive service scholarships such as HPSP [11].

Other issues to consider when evaluating physician compensation include gender wage gaps, which have been noted to affect women in all professions, including medicine [12]. Recent studies show that male physicians earn higher salaries than female physicians after adjusting for specialty, academic rank, research time, publications, and leadership roles [13]. Since military physician pay derives from rank, time in service, and incentive pay, gender pay gaps observed in the civilian sector [14] should be minimal in the military. However, data are not readily available comparing promotion rates by gender within the military Medical Corps and the MGMA data available did not reflect pay by gender. Additionally, physician compensation may not only affect physician recruitment and retention; it may also impact physician practice and potentially patient outcomes. Previous studies have shown mixed results related to physician compensation and patient outcomes [14-15]. These studies found improved patient compliance, increased continuity of care, and more fee-for-service payment visits than salaried physicians [14]. Other studies have not found an association between payment models and healthcare use, quality, and cost [14]. Further studies in this area should be conducted to assess patient compliance and outcomes in the Military Healthcare System.

## Conclusions

Our study suggests that the widely accepted belief of higher military physician compensation in residency balancing the lower military physician pay after training may be true for military physicians who attended USUHS and/or avoided significant educational debt accrual. However, direct comparisons of military and civilian attending compensation show significant negative pay differentials for military physicians, especially in surgical fields. As the U.S. Department of Defense consolidates medical care under the Defense Health Agency, our physician compensation analysis in the civilian and military pay sectors is particularly timely. Current plans to shift active duty physicians to traditional operational and wartime specialties could result in higher physician salary costs, as civilian physicians filling roles previously held by active-duty physicians would expect salaries commensurate with their civilian sector counterparts.
